# Investigation of the Antibacterial Activity of ZnO-Loaded Alginate/Hyaluronic Acid Aerogels for Wound Dressing Applications

**DOI:** 10.3390/polym17040506

**Published:** 2025-02-15

**Authors:** Tamara Athamneh, Alaa Abuawad, Tasneem Odat, Areen Alshweiat, Rana Obaidat, Farah Bani Yaseen, Mohammad A. Al-Najjar, Raghad Garafat, Razan Altarabeen, Irina Smirnova, Pavel Gurikov

**Affiliations:** 1 Institute of Nanotechnology, Jordan University of Science and Technology, Irbid 22110, Jordan; tkathamneh@just.edu.jo (T.A.); taodat20@nano.just.edu.jo (T.O.); ffbaniyaseen21@nano.just.edu.jo (F.B.Y.); rygrafat20@nano.just.edu.jo (R.G.); 2 Department of Pharmaceutical Sciences and Pharmaceutics, Faculty of Pharmacy Applied Science Private University, Amman 11931, Jordan; a_abuawad@asu.edu.jo (A.A.); moh_alnajjar@asu.edu.jo (M.A.A.-N.); 3 Department of Pharmaceutics and Pharmaceutical Technology, Faculty of Pharmaceutical Sciences, The Hashemite University, Zarqa 13133, Jordan; areen.alshweiat@hu.edu.jo; 4 Department of Pharmaceutics and Pharmaceutical Technology, Faculty of Pharmacy, The University of Jordan, Amman 11941, Jordan; r.obaidat@ju.edu.jo; 5 Institute of Thermal Separation Processes, Hamburg University of Technology, Eissendorfer Strasse 38, 21073 Hamburg, Germany; r.altarabeen@tuhh.de (R.A.); irina.smirnova@tuhh.de (I.S.); 6 aerogel-it GmbH, Albert-Einstein-Str. 1, 49076 Osnabrück, Germany

**Keywords:** antibacterial aerogels, wound healing, zinc oxide nanoparticles, hyaluronic acid-alginate composite

## Abstract

The prevalence of bacterial infections in wounds is a significant challenge to successful wound healing. This study investigates the antibacterial effect of hyaluronic acid and alginate aerogel loaded with zinc oxide nanoparticles as a potential dressing for wound healing. The aerogel composite was synthesized via supercritical gel drying and characterized by scanning electron microscope, Fourier transform infrared spectroscopy, and nitrogen porosimetry. The absorptivity of the prepared aerogel was evaluated, as well as the antibacterial activity, which was evaluated against common wound pathogens, including *Staphylococcus aureus* and *Escherichia coli*, using the agar diffusion method. The results show the effective antibacterial properties of the prepared hydrogel and aerogel. Furthermore, the results show water absorption ability of 5791 and 1585% for loaded and unloaded aerogels, respectively. The ZnO released from the aerogel exhibited a rapid release followed by a slow and sustained release. These findings highlight the potential of aerogels based on hyaluronic acid and alginate and loaded with zinc oxide nanoparticles as an innovative antibacterial wound dressing material, which is expected to improve wound healing and reduce the risk of bacterial infections.

## 1. Introduction

Skin is the primary defense mechanism of the body, which protects against the external environment and microorganisms [1]. It consists of three layers: (i) epidermis, (ii) dermis, and (iii) hypodermis. The epidermis is the outermost layer of skin and has no blood vessels; it sits above the dermis. The dermis makes up most of the skin and contains collagen, elastin, and glycosaminoglycans (GAGs). It contains fibroblast cells that attach to collagen fibers and blood vessels. The hypodermis is the innermost layer of skin, consisting mainly of loose connective tissue and fat, and serves to insulate the skin from mechanical and thermal stress [2,3].

A wound can be defined as an injury, cut, or breakage of the skin. Although microorganisms are a normal part of healthy skin and wounds, the overgrowth of the existing bacteria or the formation of a biofilm can slow down wound healing [4,5]. Despite recent progress in wound management, bacterial infections caused by *Staphylococcus aureus* (*S. aureus)*, methicillin-resistant *S. aureus*, *Escherichia coli* (*E. coli*), and *Pseudomonas aeruginosa* are still common and can cause pain in patients with infected wounds [5]. Inadequate wound infection control may lead to prolonged healing and might lead to more complicated issues like bacteremia [6,7,8].

Throughout the process of wound healing, the body naturally generates exudate as a part of the healing response [9]. Nevertheless, excessive production of exudate can represent a challenge for wound management. Thus, there is a constant need in healthcare services for the creation and improvement of novel healing devices and therapeutics that offer enhanced performance [10], especially in diabetics who are more susceptible to chronic wounds and severe complications, leading to prolonged healing times, increased risk of infection, and limb amputations [11]. The main reason for chronic wounds is that the abuse and long-term use of antibiotics lead to bacterial resistance [12], which demands novel strategies to address this challenge. The research has turned to nanomaterials, especially the metallic nanoparticles (NPs) due to their unique characteristics, such as high reactivity and multiple targets on microbial cells, which created a promising alternative to traditional antibiotics [13,14,15].

Zinc oxide nanoparticles (ZnO-NPs) have an effect on a broad spectrum of Gram-positive and Gram-negative bacteria. Also, they are safe on the human body, biocompatible, and do not promote the development of bacterial resistance [16,17,18,19]. The treatment of skin ulcers should involve the use of topical materials that support tissue regeneration while controlling free radical formation [20,21]. Thus, a wound dressing material combining both wound healing properties and antibacterial activity is of great interest [22]. The integration of ZnO nanoparticles into biopolymer-based wound dressings presents a promising strategy to enhance wound healing through improved antibacterial properties and biocompatibility.

A study focused on the fabrication of zinc oxide nanoparticles (ZnO NPs) and silver nanoparticles (Ag NPs) doped with vitamins A and E, embedded in wheat gluten films. These nanocomposites exhibited significant antioxidant and antibacterial potential, making them effective for burn wound treatment. The research highlighted the biocompatibility of ZnO and its role in enhancing tissue regeneration, suggesting that such nano-based dressings could address the limitations of traditional wound care materials [23].

Another study explored polyvinyl alcohol (PVA) and chitosan-based hydrogels containing ZnO nanoparticles. The hydrogels demonstrated over 70% antibacterial activity against E. coli and S. aureus. The incorporation of ZnO improved cell viability and accelerated wound healing, indicating that these hydrogels could serve as effective wound dressings, particularly for secondary and tertiary wounds [24].

A recent study of V. Blinov et al. (2023) demonstrated the efficacy of a gel made from hydroxyethyl cellulose modified with ZnO nanoparticles in promoting skin wound healing. The gel exhibited superior regenerative effects compared to gels containing larger ZnO microparticles, highlighting the advantages of using nanoparticles for enhanced therapeutic outcomes in wound management [25].

Wound dressing is a material used to cover, protect, and isolate the wound. Ideal dressing should maintain a moist environment, promote angiogenesis, enhance epidermal migration, and provide protection against bacterial infection [26,27]. The currently used wound dressings demonstrate an inadequate ability to treat wounds because of the prolonged healing process that might cause microbial contamination and growth [28]. In recent years, there has been a growing interest in natural polymer-based hydrogels and aerogels due to their unique properties, making them potentially suitable for diverse applications. The biopolymers, such as alginate, hyaluronic acid, chitosan, and cellulose, are becoming increasingly popular in the preparation of aerogel-based composites, as they have many advantages like availability, biocompatibility, degradability, non-toxicity, and relatively low cost compared to synthetic polymers [29]. These natural polymers have already been used, particularly in biomedical industries, and have found use in drug delivery systems and wound dressings for burns and wounds [30]. Traditional wound dressings lack antibacterial properties and cannot create a moist environment, providing only a passive protective barrier effect. On the other hand, aerogel-based wound dressings have a functional effect and excellent water-maintaining abilities, making them more beneficial. Such aerogel dressings can absorb wound exudates, maintain a favorable moist environment, and exhibit good antibacterial activity, effectively promoting wound healing. Biopolymer-based aerogels in the form of particles can be particularly useful as drug delivery systems [31] as the impregnation of active substances in aerogels can improve pharmacokinetic properties such as bioavailability and control the release of the active substance from the aerogel pores. These characteristics of aerogels make them an attractive choice for wound-healing applications.

Aerogels are highly porous material with appreciable surface area that gives rise to high adsorption capacity, allowing the impregnation with a variety of substances, such as active pharmaceutical ingredients, oils, and food additives [29,30]. The novelty of studying the antibacterial activity of ZnO nanoparticles embedded in Alg-HA aerogels, as opposed to those embedded in Alg-HA hydrogels, primarily lies in their material structure and mechanisms of antibacterial action. Aerogels are characterized by an extremely high surface area, low density, and a highly interconnected porous structure [32]. These properties allow for enhanced interactions between ZnO nanoparticles and the bacterial cells. The larger surface area of aerogels provides more space for ZnO nanoparticles to interact with bacteria, potentially enhancing antibacterial effectiveness. In contrast, hydrogels have a more compact structure with water retention, which may reduce surface interactions compared to aerogels [33]. The greater surface area and porosity of aerogels can enhance the interaction surface for ZnO nanoparticles, potentially leading to improved antibacterial activity, as larger surface areas typically correspond to greater bactericidal efficacy [34].

ZnO nanoparticles are known to have antibacterial activity through mechanisms such as the generation of reactive oxygen species (ROS) and the release of zinc ions [16]. The higher porosity and surface area of aerogels can promote more efficient ROS production and ion release, improving the antibacterial efficiency of ZnO nanoparticles [34]. On the other hand, hydrogels provide a more stable, hydrated environment that may limit these interactions. Therefore, the bacterial cells are less exposed to the ZnO nanoparticles because the gel matrix partially confines the ZnO particles and inhibits their interaction with bacteria. Thus, the interaction between ZnO and the bacterial cells may be less efficient compared to aerogels [35,36].

Among many biopolymers, alginate has served as a model biopolymer for aerogel fabrication. Alginate (Alg) is an anionic biopolymer obtained from brown seaweed and demonstrates certain interesting properties in the wound healing process, such as the ability to moisturize the skin and limit bacterial infections. Alginate has excellent gel-forming properties in the presence of cations such as calcium ions, which enable the formation of a stable three-dimensional network structure [37]. This gel-forming property of alginate plays a vital role in immobilizing and trapping active ingredients within the gel structure. The gelation ability of alginate facilitates adhesion between tissue surfaces, which promotes the painless removal of dead cells and alleviates patient discomfort during dressing changes. Due to its water-holding capacity, alginate exhibits the ability to retain moisture within the aerogel. This helps in wound healing applications, as it facilitates the creation of a moist environment that promotes the healing process. Alginate-based wound dressing materials have been developed using different types of biopolymers as additives, such as chitosan and hyaluronic acid. Upon blending with alginate, such hybrid networks not only improve the physical stability of the wound dressing but also create a moist wound environment, which is beneficial for the healing process [38]. Hyaluronic acid (HA) is composed of disaccharides and consists of N-acetylglucosamine and glucuronic acid, and it has proven wound healing properties as it improves collagen production, which is responsible for skin regeneration and wound closure. Additionally, researchers have taken note of its potential in combating microorganisms, particularly its anti-adhesion and anti-biofilm properties [39,40]. Remarkably, HA is actively involved in the early stages of tissue repair and wound healing. It works together with fibrin to create the matrix that facilitates the organization of fibroblasts and endothelial cells within the injured area. The hydrophilic nature of HA softens the fibrin clot, making it more accessible for cell colonization. These cellular processes are vital in enabling tissue regeneration by enabling the construction of the newly forming tissue’s structure [41].

This work aims to investigate aerogels based on Alg and HA loaded with the ZnO-NPs as a novel wound dressing material. The optimal loading concentration of ZnO-NPs within the aerogel matrix was evaluated to achieve the highest antibacterial activity. The prepared wound dressing was characterized in terms of fluid uptake, Fourier transform infrared spectroscopy (FTIR), nitrogen porosimetry (BET surface area), scanning electron microscopy (SEM), and antibacterial activity.

## 2. Materials and Methods

### 2.1. Materials

Sodium alginate (Alg) (catalog no. 71238) was supplied by Sigma Aldrich, Munich, Germany. Hyaluronic acid (HA, sodium salt, cosmetic grade, Mw = 9.9 × 10^5^ Da) was purchased from Xi’an Trend Biotechnology, Xi’an, China; zinc oxide nanoparticles (ZnO-NPs) (544906) were supplied by Sigma Aldrich, Germany; calcium carbonate (CaCO_3_) was supplied by Magnesia, Lüneburg, Germany; ethanol of 99.8% purity was provided by Solvochem-Holland, Rotterdam, The Netherlands; carbon dioxide (CO_2_) with a purity of 99.99% was provided by the Jordanian Gas Co., Amman, Jordan; Mueller–Hinton agar (lot no. EMHT110422013) was purchased from Biolab for splendid isolation, Budapest, Hungary; Mueller–Hinton broth was supplied by Oxoid, Basingstoke, United Kingdom; Dulbecco’s phosphate buffered saline (PBS) (lot no. CP16-1019) was supplied by Capricorn scientific, Ebsdorfergrund, Germany. D-gluconolactone (GDL) was supplied by Sigma Aldrich, Germany.

### 2.2. Methods

#### 2.2.1. Stock Solutions of Alginate and Hyaluronic Acid

Biopolymer solutions were prepared as follows: 2 g of hyaluronic acid and 2 g of sodium alginate were dissolved separately in 100 mL of distilled water and mixed thoroughly using a magnetic stirrer for 24 h until a homogenous solution appeared.

#### 2.2.2. Alginate/Hyaluronic Acid Hydrogel

A certain volume of the prepared HA stock solution was mixed with an equal volume of the Alg stock solution (blade mixer, 1200 rpm, 5 min). CaCO_3_ was added to the mixture (0.183 g of CaCO_3_ for each 1 g of solid sodium alginate in the solution) and mixed vigorously to ensure homogeneous dispersing. Finally, GDL was added to the final mixture (6 g per 100 mL). Directly after the addition of GDL, fixed volumes of the final mixture (5 mL) were cast into plastic cups and kept sealed for the next day to ensure complete gelation.

#### 2.2.3. ZnO-NPs-Loaded Alginate/Hyaluronic Acid Hydrogel

ZnO-loaded hydrogels were prepared as described in Section 2.2.2 by adding ZnO-NPs to the mixture of Alg and H to reach a concentration of 5, 10, or 20 mg/mL. All subsequent steps were as described above.

#### 2.2.4. Preparation of Aerogels

**Figure 1:**
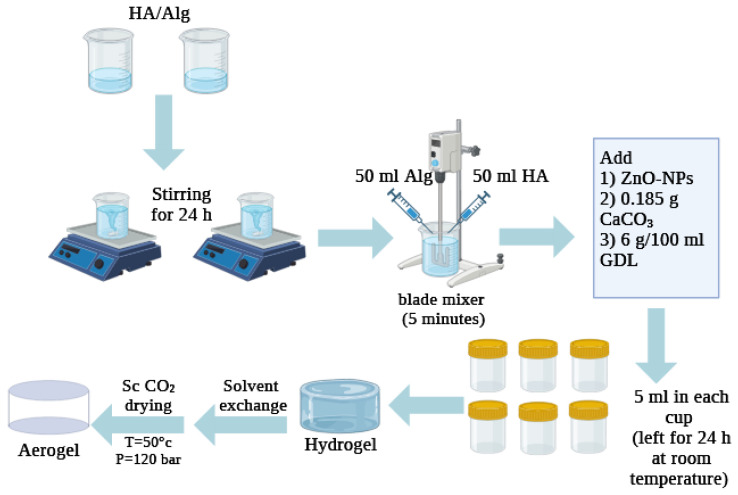
Scheme illustrating the main steps of preparation of HA/Alg composites with ZnO-NPs.

Prior to supercritical drying, hydrogels were subjected to a solvent exchange with ethanol to remove water from the gel network. The hydrogels were stepwise emerged in water/ethanol mixtures (70, 90, and 100% *v*/*v*), each step lasted 24 h. Solvent exchange started with a high concentration of ethanol to avoid the extraction of HA by the alcohol–water mixture [37]. The resulting alcogels were dried with supercritical CO_2_ at a constant temperature of 50 °C and a pressure of 120 bar. The continuous flow of CO_2_ (20–80 g/min) was set until complete extraction of ethanol was completed (~3 h), followed by a slow depressurization of the autoclave (1–3 bar/min). Once the ambient pressure was reached, the autoclave was opened, and the samples were collected. The main steps of preparation of HA/Alg composites with ZnO-NPs are summarized in Figure 1.

### 2.3. Release Studies of ZnO

The released quantity of ZnO from the nanoparticles embedded in prepared aerogel and hydrogel across cell nitrate membrane was studied using jacketed Franz diffusion cells (PermeGear, Hellertown, PA, USA) with an 8 mL receptor volume). The cellulose nitrate membraneof 25 mm thickness ((Medicell Membranes Ltd., London, UK)) was placed between the donor and acceptor compartments, which were securely clipped together. ZnO-NP loaded in HA/Alg aerogels were positioned on the upper part of the donor compartment. On the other hand, the receptor compartment contained 12 mL of phosphate-buffered saline (PBS) at a pH of 7.4 that was equilibrated to 37 °C and stirred at 600 rpm.

At specified time points of 0.25, 0.5, 0.75, 1, 2, 3, 4, 5, 6, and 24 h, an aliquot of 0.5 mL was withdrawn from the receptor compartment and replaced with the same volume of fresh preheated medium. The concentration of ZnO ($c_{ZnO}$) from the aliquots was measured spectrophotometrically (Shimadzu UV/VIS Spectrophotometer, Kyoto, Japan) at λ = 275.5 nm. The calibration curve was taken in the concentration range of 0.188–3 μg/mL. The calibration curve was linear throughout the whole range tested and was described by the equation $A=3.5007c_{ZnO}+0.0099$ (R^2^ = 0.9984, where A is absorption). The limit of detection and the limit of quantification for ZnO were 0.006 and 0.017 μg/mL, respectively. All experiments were performed in triplicate.

### 2.4. Antibacterial Activity Study

The antibacterial activity was carried out using the disc diffusion method against Gram-positive bacteria (*S. aureus*; ATCC25923) and Gram-negative bacteria (*E. coli*; ATCC14169) [42]. The two stains were grown separately on sterilized Mueller–Hinton broth (10 mL) and incubated at 36 °C for 24 h. When the optical density of the bacterial suspension reaches 0.08–0.10 at 600 nm, 100 µL was spread on a Mueller–Hinton agar plate using a sterilized disposable spreader. After spreading the bacteria, the plates were left for 5–10 min, and then the aerogel disks were placed onto the agar plates [43]. Three different ZnO-NPs-loaded aerogels (with ZnO-NPs concentrations of 5, 10, and 20 mg/mL) were studied for their antibacterial activity against *S. aureus* and *E. coli* strains and compared to ZnO-free samples. Each test was performed in triplicate, and the average inhibition zone was measured after 24 incubations (at 37 °C).

### 2.5. Aerogel Characterization

To determine the absorption capacity of the prepared aerogel, a 27 mm diameter sample of aerogel was placed in a beaker containing 200 mL of phosphate buffer saline (pH 7.4). The aerogel pieces were taken out after 0.5, 1, 2, 4, 8, and 24 h and weighed (excess buffer on the surface was dabbed away with filter paper). This mass was then compared to the original dry mass to determine the fluid uptake:

$$\mathrm{Fluid}\;\mathrm{uptake}\;(\%)=\frac{m_{s}-m_{d}}{m_{d}}\times 100\%$$

where $m_{s}$ and $m_{d}$ are the masses of swollen gel and dry aerogel. The test was undertaken in triplicate.

Scanning electron microscopy (SEM) was performed to study the morphology of aerogels and to estimate their pore size. Platinum sputtering 7 nm thickness was performed for each sample. The analysis was performed using Quanta FEG SEM 450 (ThermoFisherScientific, Brno, Czech Republic), operated at 10 kV. FTIR spectrum of samples was recorded using an Alpha FTIR spectrometer to determine the chemical functional groups in the samples. All the spectra were recorded with a resolution of 1 cm^−1^ within the range of 300–4000 cm^−1^. The specific surface area of pristine and ZnO-loaded HA/Alg aerogels was evaluated from N_2_ adsorption isotherms (Quantachrome Corporation, 360 Engineering, Golden, CO, USA) using Brunauer–Emmett–Teller (BET) method.

## 3. Results

**Figure 2:**
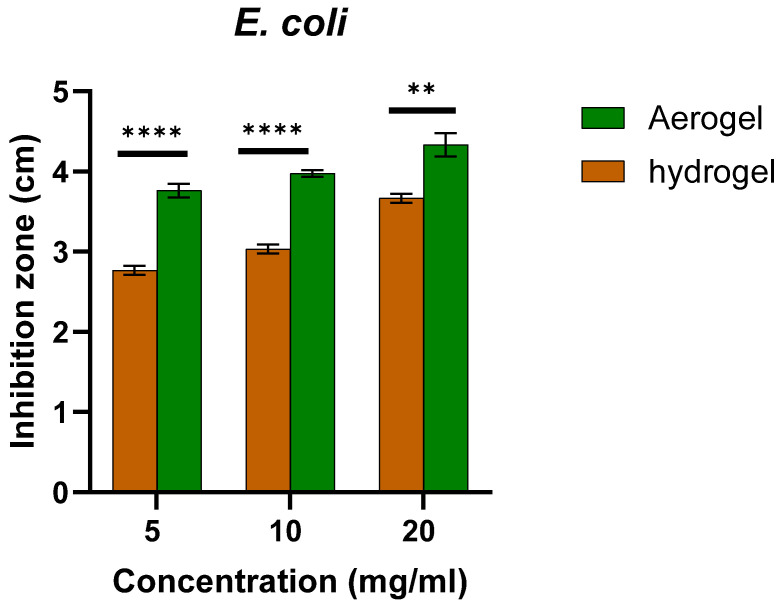
The antibacterial activity of ZnO NPs-loaded hydrogel and ZnO NPs-loaded aerogel against *E. coli* in mean of inhibition zone. ** means *p* ≤ 0.01, **** means *p* ≤ 0.0001. Error bars represent the standard deviation (n = 3).

**Figure 3:**
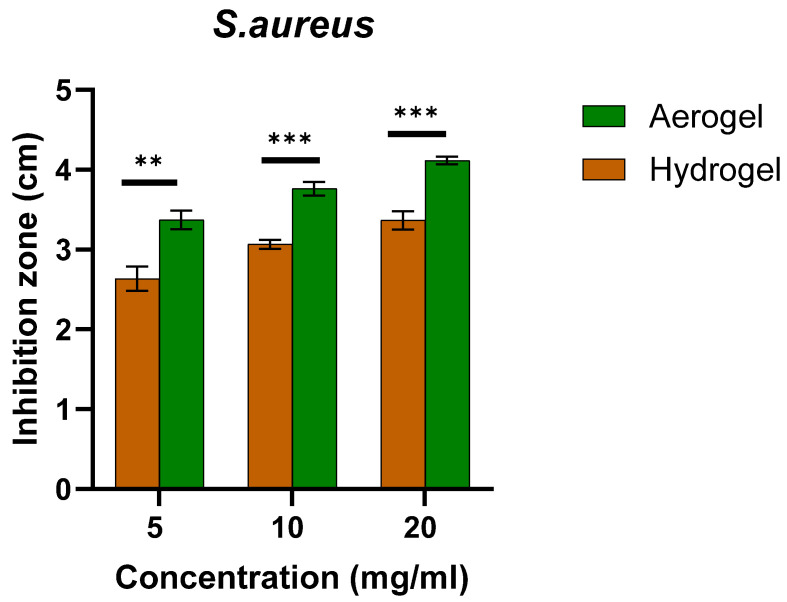
The antibacterial activity of ZnO NPs-loaded hydrogel and ZnO NPs-loaded aerogel against *S. aureus* in mean of inhibition zone. ** means *p* ≤ 0.01, *** means *p* ≤ 0.001. Error bars represent the standard deviation (n = 3).

The zone of inhibition of aerogels and hydrogels, both ZnO-free and loaded ones, was measured after 24 h incubation after calculating the average of the three readings of the clear area diameter for each concentration (Figure 2 and Figure 3). The results demonstrate a clear antibacterial effect of the ZnO-loaded aerogels and hydrogels. The antibacterial effect was observed to increase with increasing the ZnO-NPs concentration. The highest antimicrobial activity against *S. aureus* and *E. coli* for both hydrogel and aerogel samples was at 20 mg/mL ZnO-NPs. However, no zone of inhibition was shown for either unloaded hydrogel or aerogel. It should be noted that there are statistically significant differences between the antibacterial activity of ZnO-loaded hydrogels and aerogels (two-sample *t*-test, two-tailed *p ≤* 0.0017). At all loadings, the aerogels demonstrated superior antibacterial activity compared to the corresponding hydrogels.

**Figure 4:**
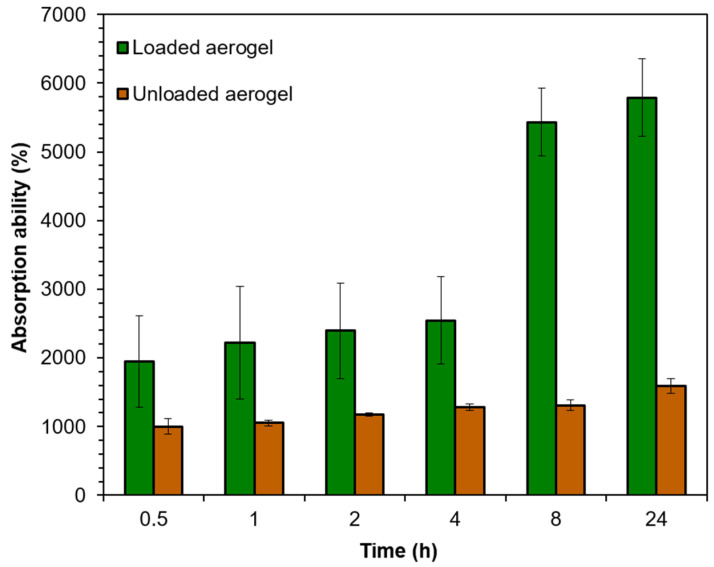
Results of the fluid uptake ability of HA/Alg ZnO-loaded (20 mg/mL) and ZnO-free aerogel.

To evaluate to what extent the developed aerogels are suitable as wound dressing for highly exuding wounds, the fluid uptake was measured for ZnO-loaded (20 mg/mL) and ZnO-free aerogels (Figure 4). The pristine Alg/HA aerogel demonstrated a quick fluid uptake of 1000% after the first 30 min, with a sustained increase up to 1585% after 24 h. These values can be translated into a fluid holding capacity of 10–15.85 g fluid per 1 g of aerogel. The presence of ZnO nanoparticles significantly enhanced the short- and long-term fluid uptake. This might be referred to as the high hydrophilicity of ZnO, which is due to the presence of hydroxyl groups on its surface. The loaded aerogel samples have an absorption ability of about 1951% after 30 min and 5791% after 24 h, showing a holding capacity of 19.5–57.5 g fluid per 1 g of aerogel.

**Figure 5:**
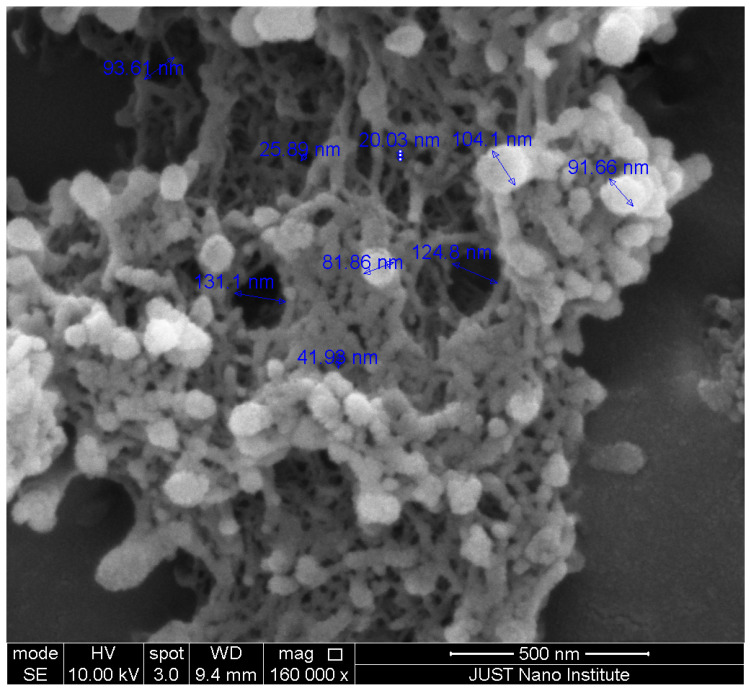
SEM image of Alg/HA aerogel loaded with ZnO-NPs (in a concentration of 20 mg/mL).

Inspection in SEM micrographs (Figure 5) revealed the presence of ZnO nanoparticles, which are incorporated into a highly porous web-like network typical for polysaccharide aerogels [44]. The open porosity is with a pore volume of 2–50 nm, also revealed by measuring the specific surface area, which amounts to 446 and 350 m^2^/g for ZnO-free and ZnO-loaded (20 mg/mL) aerogels, respectively.

**Figure 6:**
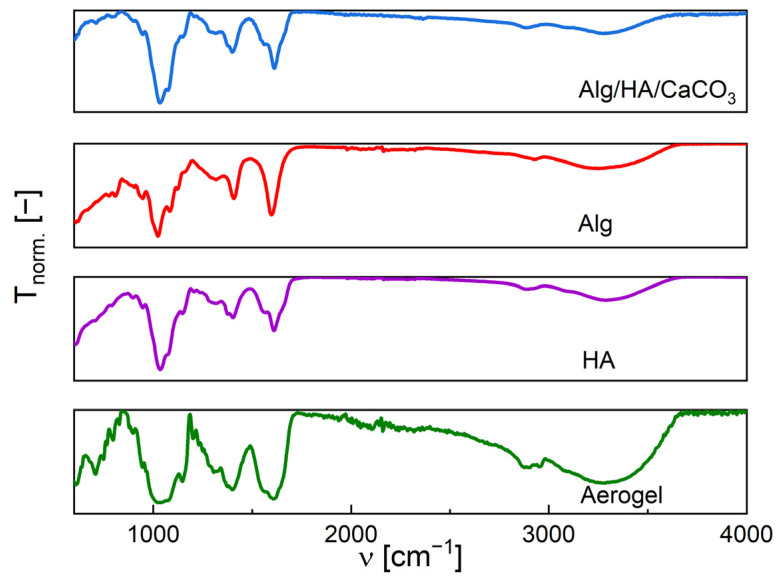
FTIR spectra of Alg/HA/CaCO_3_ physical mixture, plain Alg, plain HA, and Alg-HA aerogel loaded with ZnO-NPs (20 mg/mL). The transmittance is expressed as normalized values.

Figure 6 compares FTIR spectra of starting HA and Alg powders, the physical mixture Alg + HA + CaCO_3,_ and the ZnO-NPs-loaded aerogel. The spectrum of HA shows a broad peak around 3267 cm^−1^, indicating the asymmetric stretching of OH groups. The peak at approximately 1605 cm^−1^ with a shoulder at 1564 cm^−1^ corresponds to the C=O stretching of the amide group, while the peak at 1400 cm^−1^ is associated with the symmetric stretching of the carboxyl group (COO). Similarly, for starting Alg powder, a broad peak at 3287 cm^−1^ can be assigned to the O–H stretching vibration, while the peak at 1602 cm^−1^ corresponds to the C=O asymmetric stretching of the carboxyl group. Additionally, the peak at 1405 cm^−1^ represented the symmetric COO stretching vibration.

**Figure 7:**
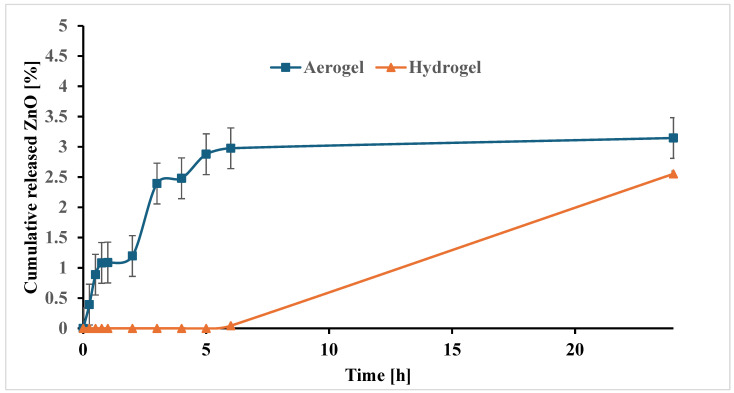
Cumulative release of ZnO from nanoparticles embedded in aerogel and hydrogel matrices (n = 3 ± SD).

The release behavior in Figure 7 showed the cumulative ZnO release from the nanoparticles embedded in hydrogel and aerogel. A biphasic release pattern was observed for aerogel that started from the first 15 min, followed by a slow and sustained release of ZnO over a 24 h period. On the contrary, the release from the hydrogel started after 5 h. After 24 h, aerogel showed a superior release of ZnO that reached 3.15% ± 0.024 compared to 2.56 ± 0.1% for the hydrogel.

## 4. Discussion

To the best of our knowledge, no direct comparisons of antimicrobial activity between aerogels and parent hydrogels have been published so far. One plausible reason for the observed differences may be attributed to the creation of a water deficit environment around aerogel due to its high affinity to water when compared to “water-saturated” hydrogel (see below results on fluid uptake). As for the specific effect of ZnO-NPs, Ahmad et al. (2022) demonstrated the efficacy of ZnO NPs (<100 nm) on Gram-positive and Gram-negative infectious strains (inhibitory zones of 1.6–2.1 cm) [45]. The antibacterial test in this study revealed a synergetic effect of the Alg/HA matrix in inhibiting bacterial growth and preventing infections. Previous studies also confirm that ZnO NPs incorporated into Alg hydrogels displayed decreased bacterial growth compared to Alg-only hydrogels, particularly against *Staphylococcus epidermidis* and *Escherichia coli* [17,46].

Jones, Grey, and Harding (2006) stated that the ability of alginates dressing to absorb 15 to 20 times their weight of fluid, makes them suitable to be used in highly exuding wounds [47]. Moreover, Kusworo, Aryanti, and Dalanta (2021) found that the addition of ZnO nanoparticles increased the porosity of the prepared membrane and successfully increased the hydrophilicity, consequently increasing the water uptake ability and also increasing the tensile strength of the modified membrane [48]. The current findings reveal the potential of the loaded aerogel to effectively manage moisture levels and consequently enhance the wound healing process.

The result for ZnO-free aerogel agrees with the study by Athamneh et al. (2019), which reported BET surface area of hybrid Alg/HA aerogels in the range 450–610 m^2^/g [37]. The drop in the surface area for ZnO-loaded aerogel cannot be explained by an increase in the sample weight since the ZnO-NPs also carry a certain surface area. From the supplier specification, the BET surface of pristine ZnO-NPs is in the range of 10–25 m^2^/g. Given the density of the bulk ZnO of 5.61 g/cm^3^, the nanoparticle size can be estimated to be 21–53 nm (assuming ideal sphericity; roughly in agreement with Figure 5). The specific surface area of a composite aerogel $S_{A+B}$ (with no synergy effects) can be estimated as follows:

$$S_{A+B}=\frac{S_{A}w_{A}+S_{B}w_{B}}{w_{A}+w_{b}}$$

where $S_{A}$ and $S_{B}$ are the specific surface areas of individual components A and B, and $w_{A}$ and $w_{b}$ are their weight fractions ($w_{A}+w_{b}=1$) [49].

The weight ratio between biopolymers (component A) and ZnO (component B) is fixed at 1:1 (g/g) (see Section 2.2.1, Section 2.2.2 and Section 2.2.3), given for the specific surface of the ZnO-loaded aerogel a narrow range of 228–236 m^2^/g. This estimation is lower than the experimental value, suggesting a certain synergetic effect of the addition of ZnO. The nature of the effect requires a well-conceived experimental design and is out of the scope of the current study. The following factors can, however, be surmised to explain the experimental result: (1) ZnO-NPs can be partially dissolved upon addition of GDL, as Zn^2+^, not ZnO is a thermodynamically stable form at pH < 7; (2) released Zn^2+^ ions form complexes with alginate, alternating the crosslinking degree of the resulting gel; (3) partially dissolved ZnO-NPs do not contribute to the overall mass of the sample, because they can be washed out during the solvent exchange [50].

Comparing the FTIR spectra of the physical mixture Alg + HA + CaCO_3_ with the corresponding aerogel, only minimal changes can be observed: two additional peaks appeared in both the physical mixture and aerogel at 852 cm^−1^ and 700 cm^−1^, which corresponds to the CO_3_ bending and stretching vibration band. Only slight differences can be noted in the width and intensity of the COO bands. The FTIR spectrum of ZnO-loaded Alg-HA aerogel shows a slight shift of the broad peak (3200–3400 cm^−1^) to 3305 cm^−1^, probably pointing to new interactions between –OH moieties and ZnO nanoparticles. The shoulder at 1564 cm^−1^, indicating the amide C=O asymmetric stretching, was no longer present. This observation could be because of the dilution of HA within the aerogel structure or the formation of hydrogen bonds between the two polymers. The peak at 450 cm^−1^ is due to the Zn-O band of the zinc oxide nanoparticles.

These results match the data reported in the literature by Athamneh et al. (2019) for zinc-free HA and Alg powders [37]. Handore et al. (2014) demonstrated that the appearance of approximately the same abovementioned peak of ZnO-NPs at around 457 cm^−1^ corresponds to the characteristic absorption of the Zn-O bond [51]. Another study by Noah et al. (2017) demonstrated the appearance of approximately the same abovementioned peak of CaCO_3_, around 712 cm^−1^ and 875 cm^−1^ [52].

Wound dressing should be applied and changed every 24 h. Therefore, the formulation should guarantee the release of an adequate amount of antibacterial agents within this time. A significant difference between aerogel and hydrogel in the release of ZnO from the embedded nanoparticles was observed. Aerogel showed a burst release, which could be related to the ZnO nanoparticles residing outside the pores in the aerogel, as the nanoparticles trapped inside the pores of the aerogel would probably take a longer time to release [53].

The release was sustained over 24 h due to the unique structure of the aerogel, with its high porosity and interconnected voids that allow for the diffusion of nanoparticles. On the other hand, the release of ZnO from the embedded particles in hydrogel started after 5 h. The long lag time observed in hydrogel could be attributed to stronger physical binding of the nanoparticles to the surface of the hydrogel. This immobilization within the hydrogel matrix was shown to slow down the initial release of the zinc oxide from the nanoparticles and result in a somewhat lower release after 24 h (3.15% vs. 2.56% for aerogel). The low release could be anticipated due to the strong attachment of ZnO nanoparticles to the –OH moieties of HA and alginate, as indicated by the FTIR analysis [17,53,54].

The current findings emphasize the advanced properties of Alg- and HA-based dressings containing ZnO NPs. The enhanced fluid uptake, antibacterial effects, and improved mechanical properties position these materials as promising candidates for effective wound healing solutions. These findings are consistent with earlier research while also providing new insights into the specific interactions and benefits conferred by ZnO nanoparticles in aerogel environments.

## 5. Conclusions

A wound dressing material based on hybrid Ca-crosslinked alginate/hyaluronic aerogel loaded with ZnO-NPs was prepared and characterized. The characterization by FTIR revealed the chemical integrity of the components in the final formulation. Open porosity with a significant fraction of pores in the range of 2–50 nm (mesopores), resulting in a high specific surface area, was observed with SEM and quantified by nitrogen porosimetry. The highly porous hydrophilic matrix of aerogel loaded with ZnO-NPs possesses a high fluid uptake of 5791% and ensures the release of an adequate amount of ZnO from the embedded nanoparticles. The ZnO-NPs endow the aerogel with antibacterial activity, which is expected to effectively prevent infection and improve the wound healing process. Antibacterial properties against bacteria associated with wound infection were demonstrated. All the results demonstrate the potential of aerogels to be used as novel dressings with improved management of wound exudate and antibacterial effect. To obtain insights about the effectiveness and safety of the ZnO and other nanoparticles in actual wound healing scenarios, further in vivo evaluation of NP-loaded aerogels is desired.

## Data Availability

The original contributions presented in this study are included in the article. Further inquiries can be directed to the corresponding author.
